# Exploring the Impact of Occupational Silica Exposure Progressing to Systemic Sclerosis: A Report on the Development of Silica-Induced Systemic Sclerosis Cases

**DOI:** 10.7759/cureus.54595

**Published:** 2024-02-21

**Authors:** Mahismita Patro, Aswathy Girija, Subho Sarkar, Prasanta R Mohapatra, Rohit Shirgaonkar

**Affiliations:** 1 Pulmonary Medicine & Critical Care, All India Institute of Medical Sciences, Bhubaneswar, Bhubaneswar, IND

**Keywords:** raynaud’s phenomena, sclerodactyly, erasmus syndrome, systemic sclerosis, silicosis

## Abstract

Erasmus syndrome is an uncommon disease brought on by exposure to silica and later manifests as systemic sclerosis (SSc) with or without silicosis. The body of literature on Erasmus syndrome is scarce. Here, we report two cases of male patients presenting with SSc after silica exposure. One of the patients had worked in the steel industry, and another had worked in the sculpture manufacturing for a decade before the presentation. It is imperative to raise awareness of this uncommon illness because avoiding further exposure remains the mainstay of management. Our case reports reemphasize the importance of occupational history in all patients of SSc.

## Introduction

Silica exposure has been reported to be associated with various autoimmune disorders [1]. Systemic sclerosis (SSc) is a multisystemic autoimmune disorder characterized by progressive fibrosis of multiple organs, predominantly involving skin and vasculature. Different occupational and environmental exposures, including silica dust, have been identified in its etiopathogenesis [2]. The association of silica exposure with SSc with or without silicosis is known as Erasmus syndrome [1,3]. This entity is clinically and serologically indistinguishable from idiopathic SSc [4]. The diagnostic criteria for Erasmus syndrome rely on the American College of Rheumatology/European Alliance of Associations for Rheumatology (ACR/EULAR) diagnostic criteria for progressive SSc and definitive history of exposure to silica [5]. The occurrence of Erasmus syndrome is very rare, with few case reports available, especially in males owing to their occupational involvement in silica-associated workplaces [1,2]. This is in contrast to the higher prevalence of SSc in females. In many cases, Erasmus syndrome may be underdiagnosed when the patient clinically presents with SSc without silicosis, where the exposure history is likely to be missed. Hence, it is of utmost importance to seek a detailed history of silica exposure in all patients of SSc. Here, we present two cases of Erasmus syndrome with silica-induced SSc and silicosis.

## Case presentation

Case 1

A 34-year-old man presented with dry cough for four years and dyspnea on exertion now progressed to modified medical research council (MRC) grade 3 over the past two years. He also reported tightening of the skin of his hands and face for the last two years. He gave a history of bluish discoloration and numbness of fingers on exposure to cold water, suggesting Raynaud’s phenomena. He also complained of pain in multiple small and large joints for the same duration. He denied any muscle weakness, skin rash, or difficulty in swallowing. He denied having other comorbidities or prior tuberculosis infection. He was a non-smoker and had worked in a steel factory for 15 years. His job was to process the melting and molding of steel in furnaces. Further, he used to make the inner lining of the furnaces using natural sandstone dust (containing 70%-95% crystalline silica) that usually erodes over some days due to wear and tear. He denied using any protective gear at the workplace, resulting in intense iron and stone dust exposure. After developing a minimal cough, he left the job and has worked as a mason for the last four years. He is also exposed to silica dust during the current construction job.

**Figure 1:**
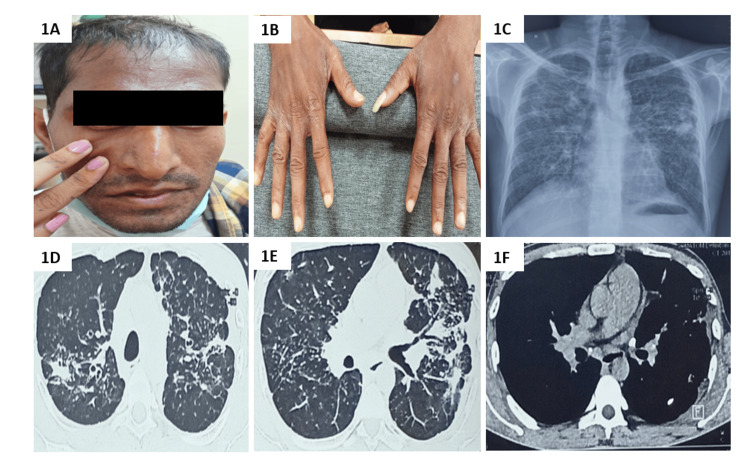
Clinico-radiological findings in Case 1 (A) Positive Ingram’s sign; (B) sclerodactyly of both hands; (C) chest radiograph in posteroanterior projection showing reticulonodular opacities in bilateral upper and mid zones; (D, E) HRCT chest lung window showing septal thickening, fibrotic opacities and conglomerated nodules in bilateral upper lobes; (F) HRCT chest mediastinal window showing calcified mediastinal lymph nodes. HRCT – high-resolution computed tomography

On physical examination, his pulse rate was 110/min, respiratory rate was 22/min, blood pressure was 116/66 mmHg, and room air oxygen saturation (SpO_2_) was 98%. The systemic examination revealed tightening of the skin over fingers (consistent with sclerodactyly), hands, and face, and digital pitted scars in the fingers. The skin over the forehead and neck showed salt and pepper-like pigmentation. The lower eyelids could not be retracted while looking for pallor (Ingram’s sign was positive) (Figures 1A-1F).

The respiratory system examination revealed bilateral normal vesicular breath sounds without any adventitious sounds. The complete blood count revealed hemoglobin of 12 g/dL, total counts of 7,300/mm^3, ^and platelet count of 2.3 lakh/mm^3^. The random blood sugar level was 84 mg/dL. The liver and kidney function tests were within normal limits. The workup for connective tissue diseases revealed positive antinuclear antibodies in nucleolar pattern and strongly positive Scl 70 antibodies. The U1 RNP was weakly positive. He fulfilled the ACR/EULAR diagnostic criteria for progressive SSc with a score of 22. The chest radiograph showed bilateral reticulonodular opacities predominantly in the upper lobe, and the nodules coalesced into masses suggestive of progressive massive fibrosis (PMF). The chest's high-resolution computed tomography (HRCT) supported the findings of PMF (Figures 1A-1F). The spirometry showed a restrictive defect with forced expiratory volume in first second (FEV1) of 1.41 L (48% predicted), forced vital capacity (FVC) of 1.97 L (56% predicted), and FEV1/FVC of 0.716. The diffusion capacity of the lungs for carbon monoxide (DLCO) was severely reduced at 3.97mL/min/mmHg (14% predicted). Transthoracic echocardiography was normal. Based on the typical clinical-radiological findings, history of significant exposure to silica, and strongly positive Scl 70 autoantibodies, a diagnosis of Erasmus syndrome with silicosis and PMF was made. The patient was started on immunosuppression with prednisolone 20mg daily and mycophenolate mofetil 500mg twice daily. He was also offered supportive care with nifedipine 20mg twice daily, omeprazole, and skin care and advised to change his job at the earliest.

Case 2

**Figure 2:**
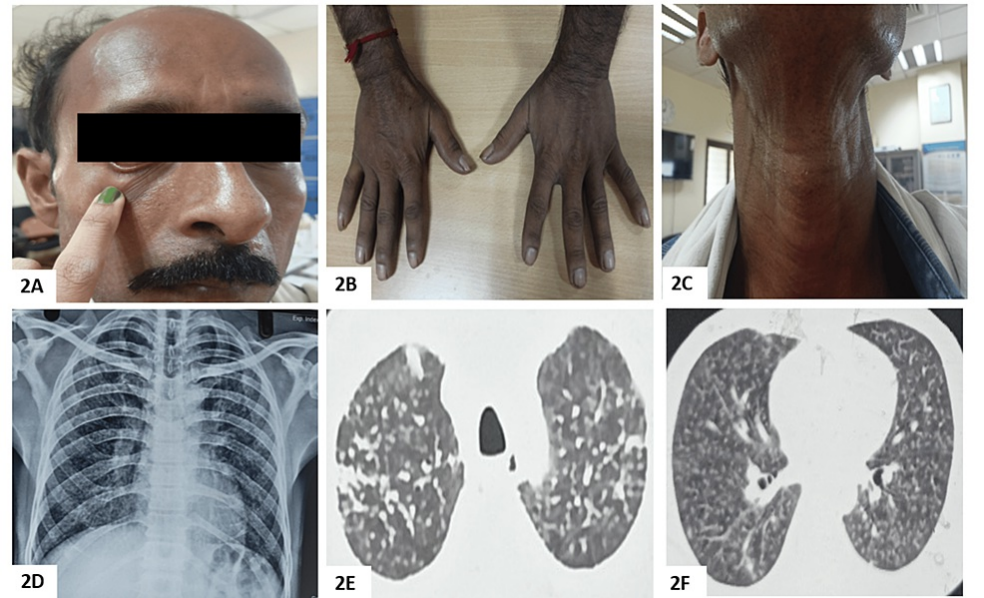
Clinico-radiological findings in Case 2 (A) Positive Ingram’s sign; (B) sclerodactyly in both hands; (C) positive Barnett’s sign; (D) chest x-ray in posteroanterior projection showing diffuse nodular opacities bilaterally in all lung zones; (E, F) HRCT chest lung window at different levels showing randomly distributed nodules in bilateral lungs. HRCT – high-resolution computed tomography

A 45-year-old man presented with complaints of cough with scanty sputum and dyspnoea of mMRC grade 2 for three months. He also complained of skin tightening and bluish discoloration and numbness of fingers on exposure to cold (Raynaud’s phenomenon). There were no other respiratory or systemic symptoms. He denied having any comorbidities or prior tuberculosis. He was a non-smoker. He was engaged in sculpture-making from stones for the past 12 years. His job included grinding and cutting stones without any protective measures, leading to unrestricted exposure to fine stone dust. For the last three months, he had stopped going to work because of his symptoms. Physical examination revealed a respiratory rate of 22/min, pulse rate of 88/min, blood pressure of 128/78 mmHg, and SpO_2_ of 90% at room air. The patient had sclerodactyly, skin thickening over the face and neck, salt and pepper pigmentation on the scalp, atrophy of mentum, and clubbing. The Ingram’s sign was positive. There was ridging and tightening of the skin of the neck on extending the head (positive Barnett’s neck sign) (Figures 2A-2F).

The chest examination revealed bilateral fine end-inspiratory velcro crepitations. The routine blood investigations were all within normal limits. The antinuclear antibodies were positive in the nucleolar pattern. The anti-Scl 70 and anti-centromere antibodies were positive. He satisfied the ACR/EULAR criteria for progressive SSc with a score of 19. The chest radiograph and HRCT chest showed randomly distributed nodules in bilateral lungs, which was similar to six months ago (Figures 2A-2F). The sputum acid-fast bacilli and cartridge-based nucleic acid amplification tests (CBNAAT) were negative. The spirometry showed a restrictive defect with an FVC of 78% (2.2L), FEV1-86%(2L), and FEV1/FVC-0.90. The patient could not perform the DLCO maneuver. Based on the occupational history, typical clinico-radiological picture, and serology, Erasmus syndrome with silicosis was diagnosed. The patient was given supportive treatment with skin care, omeprazole prophylaxis, and prednisolone 20mg once daily.

## Discussion

Silicosis is the most common and preventable occupational lung disease caused by inhalation of crystalline silica. Silicosis is a gripping disease associated with high mortality and morbidity due to its irreversible and progressive nature. The disease progresses even after the stoppage of the exposure [1,6]. Three different clinical patterns have been described based on the exposure history and onset of symptoms: accelerated, acute, and chronic silicosis. The radiological spectrum includes ground-glassing, centrilobular or random nodules, fibrotic opacities, egg-shell calcification of lymph nodes, and conglomerated masses representing PMF [7]. Apart from the respiratory effects, silica exposure has also been associated with various autoimmune diseases like SSc, systemic lupus erythematosus, rheumatoid arthritis, dermatomyositis/polymyositis, systemic vasculitis and mixed connective tissue disorder [1,8]. The association of silica exposure and SSc with or without silicosis has been termed Erasmus syndrome [1,3,9]. Erasmus first noticed this association in 1957 among a group of gold miners [3]. There were few case reports published subsequently showing the rarity of this disease. A study on a large Brazilian cohort of 947 SSc patients found Erasmus syndrome in only 0.9% of cases [10].

Silica exposure is commonly associated with occupations such as sandblasting, mining, stone cutting, granite quarrying, and silica flour packing. There are also indirect exposures to silica in certain other industries like the manufacturing industry, especially the casting and forging (iron and steel) industry [6,7]. The silica exposure history may be missed unless the work details are asked. Our second case gave an evident history of stone cutting. The first patient was a steel factory worker, but an in-depth inquiry revealed intense exposure to stone dust. Despite various government regulations health protection in most of these industries is still ignored. Also, the workers are not aware of the possible consequences of such occupational exposures till they see themselves or their co-workers diagnosed with end-stage disease. Our first case, even after developing symptoms, was continuously exposed to silica in a different occupation due to a lack of awareness.

The pathogenesis of silica exposure leading to autoimmunity has been poorly understood and presumed to be similar to silicosis. Inhaled silica gets deposited at terminal bronchioles and alveoli levels and is engulfed by the alveolar macrophages, leading to inflammatory reactions. The macrophage activation releases various inflammatory cytokines and free radicals. The fibrosing cytokines such as IL-1, IL-6, TNF, transforming growth factor (TGF)-alpha, and epidermal growth factor alpha stimulate uncontrolled collagen production and deposition in various tissues. The inflammatory cascade continues even after the death of silica-containing alveolar macrophages as the released silica particles are re-engulfed by other macrophages. Also, silica causes immune dysregulation via exposure of target epitopes for autoantibody production after silica-induced macrophage apoptosis. This eventually results in cutaneous sclerosis, vascular occlusion, and pulmonary fibrosis [1,9].

The clinical presentation of Erasmus syndrome in the absence of silicosis is similar to idiopathic SSc except for a different gender predilection. Erasmus syndrome is common in males due to its occupational association in contrast to idiopathic SSc, which is common in females [9]. So, occupational history should always be sought in detail for any male with SSc. The classical dermatologic features of Raynaud phenomena, sclerodactyly, salt and pepper skin pigmentation, and digital pitted scars are similarly seen [1,3,9]. The patients with Erasmus syndrome have more severe pulmonary involvement compared to idiopathic SSc, likely due to associated silicosis in most cases [9]. Serologically, they are also associated with anti-Scl70 antibodies [1,9]. The Erasmus syndrome can be diagnosed using the ACR/EULAR diagnostic criteria for SSc and the definitive history of silica exposure. Both of our patients had classical manifestations of SSc along with silicosis that helped us to link to the occupational association for their disease.

Erasmus syndrome is incurable. The mainstay in management is support and the avoidance of further exposure. An organ-based symptomatic treatment for Raynaud phenomena, gastroesophageal reflux, and polyarthralgia should be followed. Immunosuppressants may be given to those with progressive disease or severe systemic organ involvement. Drugs like cyclophosphamide, mycophenolate mofetil, or methotrexate have been used for Erasmus syndrome, similar to the idiopathic form. The role of rituximab and antifibrotics, i.e., nintedanib and pirfenidone in Erasmus syndrome is not clear owing to the scarce literature on the same. Specialist palliative care and pulmonary rehabilitation can improve outcomes in patients with end-stage lung disease [1,9,11].

There have been various compensation programs for silicosis patients. However, the programs lack uniformity and proper implementation, thus causing many patients to be unable to avail of the facility [12]. Also, a patient with Erasmus syndrome without silicosis may not benefit due to the lack of its wide recognition among clinicians. Thus, there is a dire need for the Government to take appropriate steps for workplace regulations to prevent occupational exposure and financial support of such patients owing to the enormous burden of silica exposure-related health menaces.

## Conclusions

Erasmus syndrome should be suspected in all cases of SSc, particularly when it occurs in males. Our report emphasizes the significance of eliciting a detailed occupational and environmental exposure history, as it can sometimes be overlooked. It is also advisable that those employed in occupations involving silica exposure should be screened, not alone for pulmonary symptoms, but to rule out development of Erasmus syndrome. The mainstay of management is avoidance of further exposure or usage of protective gear at workplaces and management of symptoms and SSc.
